# Intensity modulated radiotherapy (IMRT) or conformational radiotherapy (3D-CRT) with conventional fractionation for prostate cancer: Is there any clinical difference?

**DOI:** 10.1590/S1677-5538.IBJU.2018.0842

**Published:** 2019-12-17

**Authors:** Gustavo Viani, Ana Carolina Hamamura, Alexandre C. Faustino

**Affiliations:** 1 Faculdade de Medicina de Ribeirão Preto, Universidade de São Paulo - USP, Ribeirão Preto, SP, Brasil; 2 Departamento de Radioterapia, Faculdade de Medicina de Ribeirão Preto, Universidade de São Paulo, Ribeirão Preto, SP, Brasil

**Keywords:** Radiotherapy, Conformal, Prostatic Neoplasms, toxicity [Subheading]

## Abstract

**Purpose:**

To compare the treatment outcomes of a cohort of prostate cancer patients treated with conventional schedule using IMRT or 3DRT technique.

**Materials and Methods:**

Between 2010-2017, 485 men with localized prostate cancer were treated with conventional radiotherapy schedule with a total dose ≥74Gy using IMRT (231) or 3DCRT (254). Late gastrointestinal (GI) and genitourinary (GU) toxicity were retrospectively evaluated according to modified RTOG criteria. The biochemical control was defined by the Phoenix criteria (nadir + 2ng/mL). The comparison between the groups included biochemical recurrence free survival (bRFS), overall survival (OS) and late toxicity.

**Results:**

With a median follow-up of 51 months (IMRT=49 and 3DRT=51 months), the maximal late GU for >=grade- 2 during the entire period of follow-up was 13.1% in the IMRT and 15.4% in the 3DRT (p=0.85). The maximal late GI ≥ grade- 2 in the IMRT was 10% and in the 3DRT 24% (p=0.0001). The 5-year bRFS for all risk groups with IMRT and 3D-CRT was 87.5% vs. 87.2% (p=0.415). Considering the risk-groups no significant difference for low-, intermediate- and high-risk groups between IMRT (low-95.3%, intermediate-86.2% and high-73%) and 3D-CRT (low-96.4%, intermediate-88.2% and high-76.6%, p=0.448) was observed. No significant differences for OS and DMFS were observed comparing treatment groups.

**Conclusion:**

IMRT reduces significantly the risk of late GI severe complication compared with 3D-CRT using conventional fractionation with a total dose ≥74Gy without any differences for bRFS and OS.

## INTRODUCTION

Radiotherapy has played a pivotal role in the management of patients with localized prostate cancer (1). In the last decades, randomized trials and meta-analysis shows that higher doses result in better biochemical control and disease survival (2). More recently, strong evidence has supported that prostate cancer cells need more RT dose and responded satisfactorily to the higher RT dose per fraction (3). Radiation therapy has passed by a great technology advance with the development of intensity modulated radiotherapy and image guided radiotherapy (1, 4). IMRT has the capability of reducing the radiation doses on the surrounding tissues such as bladder and rectum, and consequently, it has great potential to reduce the toxicities related to the treatment (4). However, IMRT is more time consuming and expensive than 3DCRT, and although, in developing countries IMRT has been adopted as the standard radiation treatment for prostate cancer, currently, in middle-and low-income countries like Brazil, the IMRT is not covered by the public health system (5). On the other hand, there are a few studies with adequate follow-up and homogeneous group comparing these techniques (6-9).

The studies comparing these techniques have some flaws such as a heterogeneous dose, differences in the follow-up time, and limited sample size (6-8).

Therefore, the present study was designed to compare the treatment outcomes with 3DCRT or IMRT from a cohort of prostate cancer patients treated with a conventional fractionation with a radiotherapy dose ≥74Gy.

## MATERIALS AND METHODS

The present study is a retrospective cohort review from a single institution. The study enrolled 485 prostate cancers with localized disease. The study began in January 2010 and closed in January 2017.

### Evaluation

All patients before the treatment were evaluated by a full history and physical examination. Patients were classified in low, intermediate and high-risk group according to their Gleason score, T stage and initial PSA (iPSA) (10). Low risk group included patients with Gleason score <7/stage T1--T2a, and iPSA <10ng/mL. Intermediate risk included Gleason score <7, or Stage T1-T2b, or iPSA level of 10-20ng/mL; and high-risk patients with Gleason score >7, or Stage >T2b, or iPSA >20ng/mL. All patients classified as high risk were submitted to bone scans.

### Exclusion criteria

Patients with metastases, prior history of prostatectomy, chemotherapy treatment, or treated with pelvic radiation due prostate cancer were excluded of this trial. Patients during the study period submitted to hypofractionated radiotherapy were also excluded from this cohort.

### Treatment

The IMRT or 3D-CRT plan consisted of 5-7 fields to deliver a total dose ≥74Gy with 1.8-2Gy per fraction prescribed at the isodose line covering 95% of PTV. All patients were simulated on CT simulator. Patients were advised that extreme bladder or rectal filling could not be present at the time of the planning CT. An enema before the planning CT scan to empty the rectum and 2-3 glasses of water were recommended. A triangle sponge under the knees was used for all patients on the treatment planning CT. The following structures were contoured as organ at risk: femoral heads, the rectum, bladder, and the penile bulb. The contours of structures followed the recommendations of RTOG (11). The rectum was contoured from anal verge to rectosigmoid transition. The low-risk group had only prostate gland countered as clinical target volume (CTV). Intermediate and high-risk group had prostate gland plus seminal vesicles base (1cm) contoured as CTV. The planning target volume (PTV) was created with 1cm margin on the CTV except for rectal wall (7mm). The study used the following rectal dose volume histogram (DVH); V50<50%, V60<35%, V65<25%, V70<20%, and V75<15%. The bladder DVH constraints were used; V54Gy<=50%, V58Gy<=35%, V 62Gy<=25% and V 68Gy<=15%. All the treatment planning was performed by the Xio^®^ (Elekta Medical Systems) and Eclipse^®^ version 13.0 (Varian Medical Systems, Inc, Palo Alto, USA). All fields were treated daily in a megavoltage linear accelerator-6MV with multileaf collimators. The digital portal images with X-ray using bone landmarks were obtained before the treatment for all patients. Patients with no setup error on the first digital portal image were checked weekly. Patients with set-up errors on the digital portal images were checked with repeatedly imaging. Patients without set-up errors on the repeat imaging were checked by orthogonal images weekly. Patients classified as intermediate and high-risk group underwent to androgen blockage. The androgen blockage was done with goserelin acetate 3.6mg. A total of 6 and 24 months of androgen blockage (neoadjuvant, concomitant and adjuvant) were administered for patients classified as intermediate and high-risk group, respectively.

### End points

The primary endpoint of this study was to compare the clinical outcomes of prostate cancer patients treated by 3D-CRT and IMRT. The biochemical control was defined as nadir +2ng/mL, according to PHOENIX criteria (12). Late toxicity was defined as any symptom beginning after 3 months of treatment. The RTOG system was used to score the toxicity (13). The evaluation after RT included serum PSA, and documentation of treatment-related toxicity at 3-6 months for the first 5 years and annually thereafter.

#### Statistical analysis

Continuous and dichotomic variables were expressed as mean/standard deviation, and proportions, respectively. Comparison between proportions was performed with chi square test. The Kaplan-Meier method was used to estimate the biochemical control and late toxicity over the time. Log-rank test was used to estimate the proportion the event between different groups over the time. The biochemical recurrence-free survival (bRFS), distant metastases-free survival (DMFS), and overall survival (OS) were the outcomes evaluated. The intervals to PSA recurrence, metastasis, and death were all defined relative to the end of RT until the event of interest, death, or last-follow-up visit. Statistical Analysis Systems software (SPSS) was used to perform all the statistical analysis. A p value <0.05 was considered statistically significant.

## RESULTS

Between January 2010-2017, 485 patients with diagnosis of prostate cancer treated by radiotherapy fulfilled the inclusion criteria of this study. All patients were treated by radiation therapy restricted to the prostate gland ± seminal vesicles combined or not with ADT. The RT total dose ranged from 74Gy to 78Gy; 254 patients were treated with 3D-CRT and 231 with IMRT technique. Table-1 describes the patient characteristics of this cohort. The groups were homogeneous with no significant differences for risk group, age, PSA level, Gleason score, RT dose and follow-up time (p>0.05).

**Table 1:** Characteristics of prostate cancer in both treatment groups.

Group		3D-CRT 254	IMRT 231	P value
**Age**		70.5 (± 8.4)	70.9 (± 7.7)	0.578
**iPSA**		15.8 (± 18)	14.2 (± 16)	0.302
Tumor stage*				0.850
	T1a-T1c	95 (37.5)	99 (43)	
	T2a-T2c	134 (52.7)	113 (49)	
	T3a-T4	25 (9.8)	18 (8)	
**Gleason Score**				0.961
	≤6	127 (50)	123 (53)	
	7	88 (34.5)	77 (33)	
	8-10	39 (15.5)	31(13.5)	
**Risk Group**				0.472
	Low	84(33)	85(37)	
	Intermediate	93(36)	87(38)	
	High	77(31)	59(25)	
**RT dose**				0.429
	74 Gy	87(34)	71(31)	
	76 Gy	100(39)	87(37)	
	78 Gy	67(27)	73(32)	
Androgen blockage**				0.706
	Yes	161(64)	150 (66)	
	No	90 (36)	78 (34)	
**Follow up**		51 (38-72)	49 (36-69)	0.08

* One single patient from high-risk group had not information about the tumor stage.

** Four patients from IMRT group classified as intermediated or high-risk group and nine from 3DRT group did not received androgen blockage.

### bRFS, DMFS and OS

**Figure 1:**
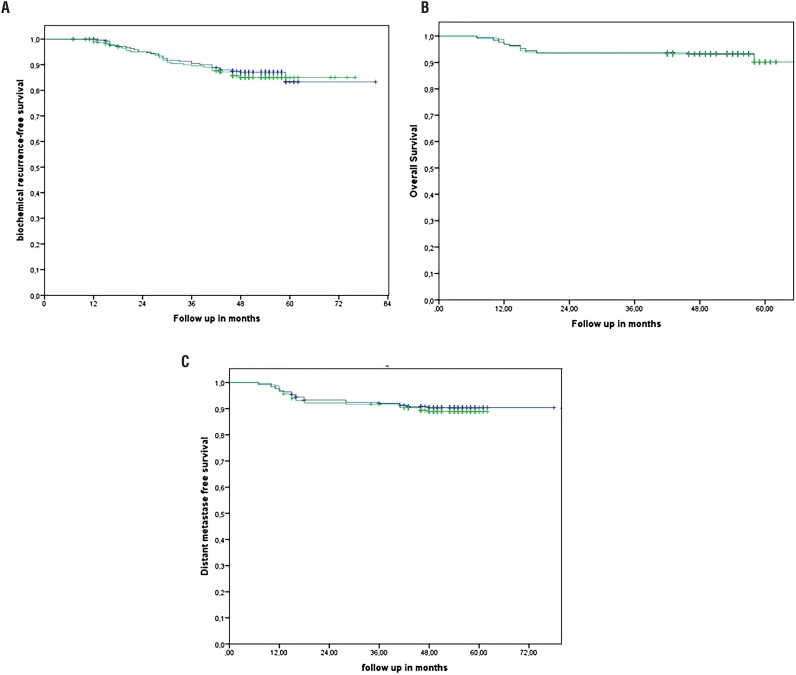
a) bRFS, b) Overall survival, c) distant metastases free survival comparing 3d-CRT versus IMRT, blue line 3D-CRT and green line IMRT.

The median follow-up time was 51 months in the IMRT group and 50 months in the 3D-CRT group. The 5-year bRFS for all risk groups with IMRT and 3D-CRT was 86.1% vs. 87.4 % (p=0.665), Figure-1a. The 5-year OS and DMFS was 92.3% and 89.9 % with no significant difference between IMRT (OS-92.5% and DMFS-89%) and 3D-CRT (OS-92.5% and DMFS-90.6%), Figures-1b and c.

**Figure 2:**
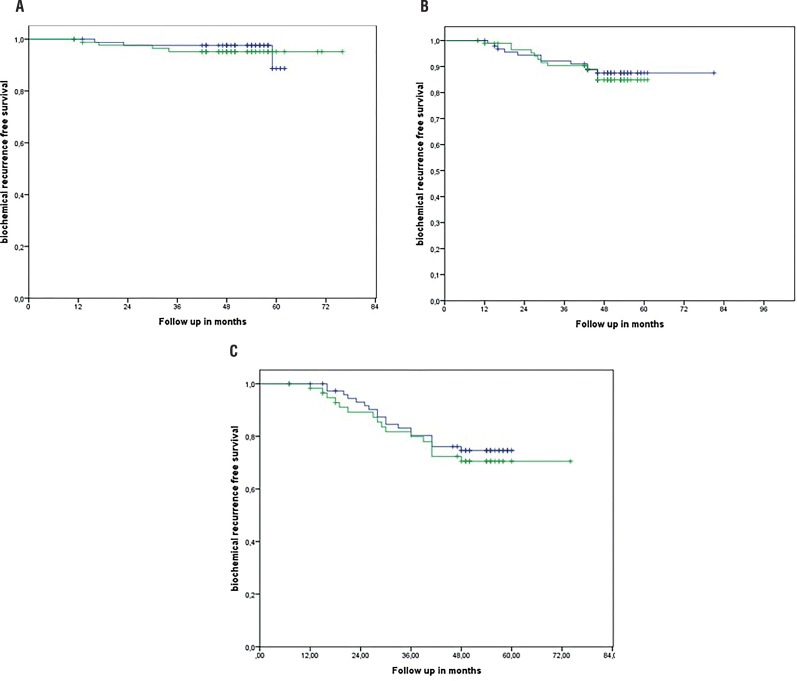
bRFS comparing 3D-CRT versus IMRT by risk group, a) low, b) intermediate, c) high-risk group.

Considering the risk-groups, no significant difference for low, intermediate-and high -risk groups between IMRT (low-95.3%, intermediate-86.2% and high-73%) and 3D-CRT (low-96.4%, intermediate-88.2% and high-76.6%, p=0.448) was observed (Figures 2a-c). During the follow-up 37 (7.6%) patients died with 22 (4.5%) died related to prostate cancer, and 49 (10.1%) patients developed metastases, as described in Table-2.

**Table 2:** Clinical outcomes by treatment group.

Variable	3D-CRT group 254 patients	IMRT group 231 patients	P value
**Death from any cause**	19	18	0.89
**Cancer death**	2	1	0.61
**Metastases**	24	25	0.63
**Biochemical recurrence**	32	32	0.68
**Disease progression**	49	45	0.95

### Late toxicity

Patients of 3D-CRT group experienced a rate of grade ≥2 late gastrointestinal (GI) toxicity of 24%, compared with a rate of 8.2% in patients treated with IMRT (0.0001). The rate of grade ≥2 late GU toxicity of 3D-CRT was 15.4% compared to 12.6% of the IMRT group (p=0.850). There were cases of grade ≥3 late GI (3) or GU (1) toxicities in 3DRT arm and no case in IMRT. All grade ≥3 late toxicities developed until 36 months from the ending of the radiotherapy. Table-3 describes the maximal late toxicity according to the RT treatment group.

**Table 3:** Maximal late toxicity comparing 3DRT vs IMRT in prostate cancer according to RTOG.

Group		Grade 0	Grade 1	Grade 2	Grade 3	Grade 4	P value
**3D-CRT (254)**							0.0001
	Gastro-intestinal	163	30	47	14	0	
		64.2%	11.8%	18.5%	5.5%		
**IMRT (231)**							
	Gastro-intestinal	159	49	19	4	0	
		68.8%	21.2%	8.2%	1.7%		
**3D-CRT (254)**							0.850
	Genitourinary	173	42	32	7	0	
		68.1%	16.5%	12.6%	2.8%		
**IMRT (231)**							
	Genitourinary	163	39	24	5	0	
		70.8%	16.9%	10.9%	2.2%		

## DISCUSSION

Over the past decade's radiation therapy has experienced a great technological advance which have allowed safe escalation of radiation dose for localized prostate cancer (1, 2, 4). Many clinical trials were conducted to test the value of dose-escalated radiation therapy (≥74Gy) compared to conventional dose (70Gy) (2). Based on those clinical trials, we can conclude that dose escalation improves the biochemical control, with an increase in the rate of late gastrointestinal toxicity and no benefit for overall survival (2).

Apart from the one trial that used proton beam therapy, all other studies used 3D-CRT to deliver a dose higher than 74Gy to the prostate gland, and IMRT was not available or allowed in none of them (2).

IMRT has been consistently associated with a lower rate of late rectal toxicity compared with 3D-CRT in patients receiving definitive RT for localized prostate cancer (4, 6-9).

In a propensity score-adjusted analysis of Surveillance, Epidemiology, and End Results from 2000 to 2009, Sheets et al. demonstrated a reduction in the late gastrointestinal toxicity due to the use of IMRT compared with 3D-CRT (14). In the present study we also confirm the better rate of late GI toxicity with IMRT over 3D-CRT using a conventional fractionation schedule with a total dose ≥74Gy. Although other single-institution studies have also reported a reduction in toxicity with the use of IMRT compared with 3D-CRT, the interpretation of their outcomes are complicated by using different radiation doses, different treatment volumes and short follow-up time (6, 8). However, even with these differences, our data also suggest that there is no difference in late GU toxicity between 3D-CRT and IMRT with total dose ≥74Gy.

The probable explanation for the absence of late GU toxicity with IMRT can be associated to the fact that the bladder neck and prostate urethra are unavoidably part of the treatment volume in both techniques. Moreover, the daily variation of the bladder filling during radiotherapy course makes difficult the translation of the dosimetric advantage with IMRT into clinical benefit over 3D-CRT. Finally, IMRT produces more heterogeneous dose within the target volume and daily set-up errors and the hot spot dose close to the urethra can contribute to this dosimetric advantage from IMRT disappear clinically when compared with 3D-CRT.

The five years bRFS for the low-(95%)/intermediate-risk group (88%) of the present study is equivalent to the results of other authors who used similar radiation total dose (15). For instance, in another cohort of Brazilian's patients with intermediate-high risk prostate cancer treated with a dose-escalation (74-80Gy) IMRT technique, the biochemical control was 89% in 5 years (15).

Currently, in developed countries IMRT is considered the standard radiation technique to treat prostate cancer. Recent surveys show that more than 90% of radiation oncologists in the USA use IMRT to treat prostate cancer (16). In opposite, developing countries like Brazil do not cover the IMRT for patients who are users of the public health system. However, even with no coverage by the public health system, some public Brazilian's institutions compromised with the medical educational and medical resident programs have the technology and offer it to treat their patients without any reimbursement for that (17). We hope that our data can contribute to further studies on cost effectiveness analysis to try to change that reality.

## CONCLUSIONS

The present series is the first homogeneous cohort of prostate cancer treated by IMRT or 3D--CRT that compares the treatment outcomes with a long-term follow-up. Our data show that both techniques with a dose between 74-78Gy produce an excellent biochemical control. However, IMRT was associated with a lower rate of late GI toxicity than 3D-CRT. In our data no significant difference was observed for late GU toxicity between the treatment techniques, we hypothesized that the inclusion of bladder neck and urethra in the target volume for both techniques and the variation of the bladder filling during the radiation course can contribute for this finding. Further studies with IGRT with cone beam CT are necessary to confirm or not this hypothesis.
